# Flow Cell Design for Effective Biosensing

**DOI:** 10.3390/s130100058

**Published:** 2012-12-20

**Authors:** Douglas J. Pike, Nikil Kapur, Paul A. Millner, Douglas I. Stewart

**Affiliations:** 1 Pathogen Control Engineering (PaCE) Institute, School of Civil Engineering, University of Leeds, Leeds, West Yorkshire, LS2 9JT, UK; E-Mail: d.i.stewart@leeds.ac.uk; 2 Institute of Engineering Thermofluids, Surfaces & Interfaces (iETSI), School of Mechanical Engineering, University of Leeds, Leeds, West Yorkshire, LS2 9JT, UK; E-Mail: n.kapur@leeds.ac.uk; 3 Institute of Membrane and Systems Biology, Faculty of Biological Sciences, University of Leeds, Leeds, West Yorkshire, LS2 9JT, UK; E-Mail: p.a.millner@leeds.ac.uk

**Keywords:** fluidics, sensors, flow cell, computational fluid dynamics

## Abstract

The efficiency of three different biosensor flow cells is reported. All three flow cells featured a central channel that expands in the vicinity of the sensing element to provide the same diameter active region, but the rate of channel expansion and contraction varied between the designs. For each cell the rate at which the analyte concentration in the sensor chamber responds to a change in the influent analyte concentration was determined numerically using a finite element model and experimentally using a flow-fluorescence technique. Reduced flow cell efficiency with increasing flow rates was observed for all three designs and was related to the increased importance of diffusion relative to advection, with efficiency being limited by the development of regions of recirculating flow (eddies). However, the onset of eddy development occurred at higher flow rates for the design with the most gradual channel expansion, producing a considerably more efficient flow cell across the range of flow rates considered in this study. It is recommended that biosensor flow cells be designed to minimize the tendency towards, and be operated under conditions that prevent the development of flow recirculation.

## 1. Introduction

Flow cells facilitate analyte delivery to biosensor surfaces for a range of applications such as detecting pathogenic bacteria [1], clinical diagnostics [2], food analysis [3], and the detection of explosives [4]. A common approach is to use flow cells with biosensors constructed upon screen printed electrodes, providing a compact analysis system applicable for both laboratory based analysis and on-site use [5–8]. Flow cells can either be purpose-built for the biosensor of interest [9] or biosensors be developed for use with a commercially available flow cell [10]. Analyte delivery may be to an initially dry flow cell [11], or to a flow cell that already contains fluid (a wet cell). The latter includes some fluid injection systems, and situations where the biosensor requires wet storage (e.g., buffer solution). It is wet cells that are specifically addressed within this paper and a fundamental requirement of these flow cells, and one frequently overlooked, is a clear relationship between the concentration of analyte in the feed solution and that within the fluid volume on which the measurement is made.

**Figure 1.**
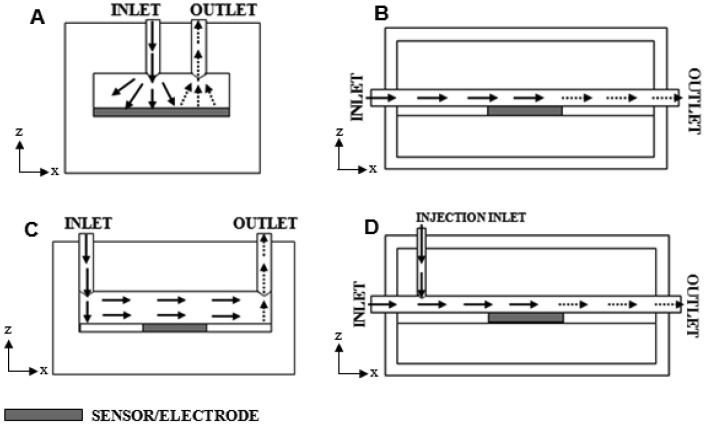
Schematic diagrams of common flow cell design types used with biosensors and other sensor types: (**A**) impinging jet direct onto sensor surface, (**B**) channel flow with embedded sensor, (**C**) impinging jet delivery with channel flow over embedded sensor, (**D**) injection into a flowing carrier channel flow

There are a vast array of designs for flow cells, but the most common systems include an impinging jet of analyte delivered directly onto the sensor surface (Figure 1(A)), analyte flow entirely parallel to a surface containing an embedded sensor (Figure 1(B)), an impinging jet delivering the analyte into a chamber containing the sensor (Figure 1(C)), or an impinging jet injecting pulses of analyte into a solution flowing across the sensor (Figure 1(D)).

**Figure 2.**
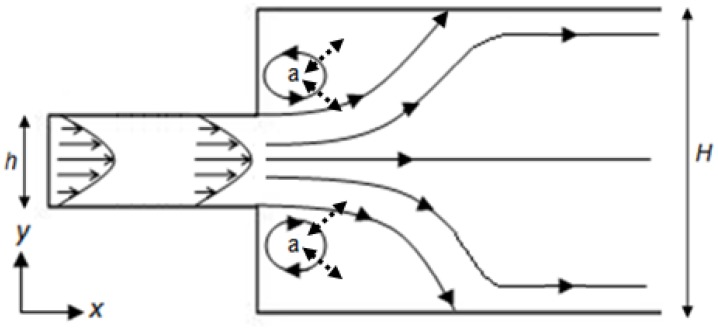
Schematic plan view of laminar flow in an open channel with a sudden expansion in width, where eddies (a) have developed. Solid arrows indicate direction of advective transport from the inlet to the outlet. Analyte transport from within developed eddies occurs only through diffusion, as indicated by broken-line arrows. Adapted from Acrivos and Schrader [12].

The flow within most flow cells will be laminar (as opposed to turbulent), that is, under a given flow rate the velocity at any given point will be constant. The Reynolds number, *Re* (*Re* = ρ*Ud*/*μ*, where *U* is the mean velocity within the flow cell, *d* is the mean width of the cell, and *ρ* and *μ* are the fluid density and viscosity), can be used to determine the status of this flow. However, even where the flow is laminar, regions of reversed fluid motion (eddies) can develop in a flow channel with an increasing channel width [12] (Figure 2). The flow patterns that exist within a wet flow cell, including the presence of eddies, determine how quickly the concentration of the analyte within the flow cell itself reaches that of the injected fluid (termed influent). The transport of analyte is controlled by two mechanisms; advection (movement in the direction of flow), and diffusion (due to concentration gradients within the fluid). Figure 2 illustrates this, within the “closed” eddies marked (a), the only mechanism for analyte transport is by diffusion, which is relatively slow; whilst outside of these eddies advective transport will be the dominant mechanism. Crucially, the precise flow pattern determines the flow volume and the flow rate (and hence time) required for the flow cell to reach the concentration of the influent. Failure to understand the behavior of the flow cell can result in an assay being made on an unknown dilution of the influent fluid.

There has been very little work published on the impact of the design and operation of flow cells on the accuracy of biosensor measurement. Rare exceptions are Cooper and Compton [13], who considered the impact of analyte diffusion to the electrode surface on biosensor response; however they assumed that flow velocity in a channel flow-cell varies parabolically with distance from the sensor surface, and thus implicitly ignored the impact of channel geometry on the flow regime. Likewise Lammertyn [14] developed a model for the reaction kinetics of a specific biosensor system. To date, there has been no systematic investigation of the impact of flow cell geometry on the analyte concentration in the active region of the biosensor cell.

Techniques for analyzing such flow systems include both computational and laboratory based approaches. Computational Fluid Dynamics (CFD) simulations provide a numerical method for predicting the flow system response to specified flow conditions and flow system design [14]. In contrast, the fluorescence and/or absorbance properties of selected fluorescent dyes have been exploited for quantitative flow analysis in laboratory based experiments [15]. The theory of residence time distribution provides a framework to describing the distribution of times taken for fluid to pass through a flow system [16], and can be applied to both the results of CFD simulation [17] and in interpreting laboratory based flow analysis experiments [18].

This work presents, for the first time, a complete and holistic application of flow analysis techniques to a biosensor flow cell. It will illustrate the relevance of fundamental flow principles and flow cell design to biosensor operation. It also investigates flow cell response to variations in design and prevalent flow conditions. Finally it presents a framework for the design and use of flow cell systems to the sensor community. This has been achieved through a combination of experiments and validated CFD simulations.

## 2. Experimental Section

This work determines (both experimentally and computationally) the efficiency of the flow cell in reaching the composition of the influent. Efficiency is assessed both in terms of the volume of influent and the time required for the process. For this study, a model flow cell has been designed to (ultimately) accommodate a biosensor constructed upon a screen printed electrode for which the working, reference and counter electrodes are contained within an 8 mm diameter circular region (e.g., the Dropsens C223AT electrode [19]).

**Figure 3.**
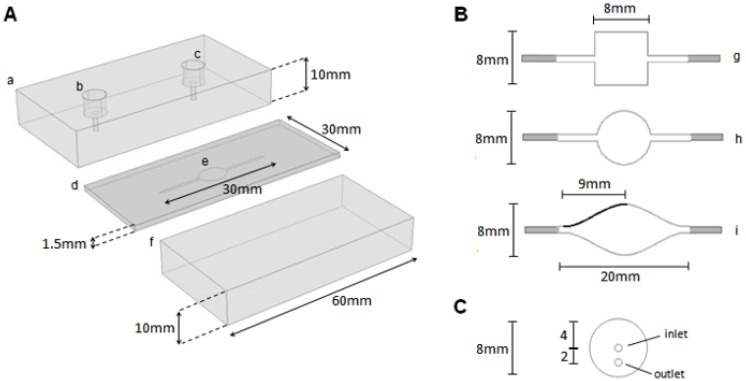
Experimental flow cell construction (**A**): (a) PMMA top block with (b) inlet and (c) outlet holes. Inlet and outlet accommodate ¼″-28 threaded nuts at the top and feature 1mm openings at the fluid inlet/outlet point; (d) PTFE gasket with (e) 30mm cut-out flow domain section; (f) PMMA base block. Bolt holes used to secure the flow cell unit are not shown for clarity. (**B**) Flow domain channel geometries: (g) square cell; (h) circular cell; (i) iCell (the heavier line of the iCell shows the curve shape upon which this flow cell is profiled); unshaded area indicates region of interest for flow analysis. (**C**) Modeled impinging jet flow cell geometry. The height of flow domains for (b) and (c) is 1.5 mm.

The entire flow chamber was 30 mm in length and consisted of small rectangular flow channels of 1 mm joining the inlet and outlet ports to a wider central region (Figure 3(A)), for which three different shapes were studied. These central region geometries were (in cross-section) a square (chamber volume 0.096 mL), a circle (0.075 mL) and a more gradually expanding and contracting geometry (0.12 mL) (Figure 3(B)). The latter, termed the iCell (due to its similarity to the shape of an eye) was developed from a 3rd order polynomial. One quarter of the profile is given by the local *x*, *y* coordinate positions (mm) *y* = (*C*_1_*x*^3^ + *C*_2_*x*^2^ + *C*_3_*x* + 0.5), (0 ≤ *x* ≤ 9) with *C_1_* = −(2*b*/*a*^3^), *C_2_* = (3*b*/*a*^2^), and *C*_3_ = 0, and subject to the size constraints given above, with *a* = 9.0 mm and *b* = 3.5 mm. This curve was reflected about the vertical (*x* = 9) and horizontal planes (*y* = 0) to give the final geometry of the central part of the flow cell. The height of the flow channel has been fixed at 1.5 mm since preliminary experiments demonstrated this to be a lower limit beyond which practical operation (e.g., bubble entrapment, filling) became difficult. A commercially available impinging jet flow cell (“FLWCL”, Dropsens, Llanera, Asturias, Spain) was also analyzed for comparison with the three channel flow cells.

### 2.1. Experimental Study

The experimental flow cell was manufactured from two transparent PMMA blocks of dimensions 60 × 30 × 10 mm with a 1.5 mm thick PTFE gasket placed between them (Figure 3(A)) into which the flow channels were cut. Six M4 bolts were used to sandwich the PTFE gasket between the two blocks thus creating a seal. Inlet and outlet holes of diameter 1 mm were drilled into the uppermost PMMA block (Figure 3(A)) to which 1/8″ OD PTFE tubing (ID 1.59 mm) was connected using threaded nuts and ferrules to ensure a robust seal. Two syringes mounted on a pair of syringe pumps (Aladdin, World Precision Instruments Ltd, Hitchen, UK) were connected to the inlet via a tee-piece to allow easy switchover between the fluid initially used to fill the cell and the influent. The distance between the outlet of the tee piece and the flow cell was minimized (55 mm) to limit mixing of these fluids outside of the flow cell. A 100 μM aqueous fluorescein sodium salt solution (C_20_H_10_Na_2_O_5_) was used as a model analyte, allowing quantification of the analyte concentration within the flow cell by measurement of fluorescence intensity. A mercury lamp (Dolan Jenner MHR 100, Boxborough, MA, USA) fitted with a fiber optic light guide was positioned to shine vertically upwards through the flow cell. A pair of bandpass filters located before (CW 490 nm ± 2 nm FWHM 10 nm ± 2 nm) and after (CW 520 nm ± 2 nm FWHM 10 nm ± 2 nm) the flow cell ensured that only light emitted by the sodium fluorescein (CW 515 nm) reached a CCD monochrome camera (Adimec 1000 m, Eindhoven, The Netherlands) attached to a monocular microscope. Images were recorded in AVI format using LabVIEW 8.0 (National Instruments, Austin, TX, USA) at a rate of 25 frames per second. Tests using solutions of different concentrations (0–100 μM) showed a linear relationship between the light intensity and the concentration of the fluorescein sodium salt solution within the flow cell.

Prior to the experiment, the flow cell was filled with deionized water. The experiment commenced with the simultaneous start of image capture and the pump containing fluorescein sodium salt solution at a predetermined flow rate (1 or 10 mL/min). Experiments were run until no change was observed between subsequent images; this took between 35 and 165 s depending on flow rate and flow cell geometry. Selected frames of the video sequence were processed using Matlab (Mathworks, Natick, MA, USA). Each pixel within the flow domain was analyzed to determine a scaled concentration, *C_ratio_*, where 0 corresponds to no analyte and 1 corresponds to the maximum concentration of analyte. For the case here, where fluorescence intensity is linearly related to concentration, the concentration ratio is related to the relative fluorescent intensity by the equation:

(1) $$C_{\text{ratio}}=\left(I-I_{0}\right)\;/\;\left(I_{\text{end}}-I_{0}\right)$$

where *I*_0_ is the background intensity of that pixel at the start of the flow experiment and *I_end_* is the fluorescence intensity of that pixel at the end of the flow experiment. In addition to determining the local concentration ratio, the mean concentration ratio within the flow cell was calculated.

### 2.2. Numerical Study

Numerically, the response of the flow cell is studied using the time-dependant advection-diffusion equation for an incompressible flow. This allows simulation of transport of a dilute species (in this case sodium fluorescein) through a solvent (water) due to advection and diffusion:

(2) $$\underset{\text{variation of concentration with time}}{\underset{︸}{\frac{\partial c}{\partial t}}}=\underset{\text{diffusion term}}{\underset{︸}{D\nabla^{2}c}}-\underset{\text{advection term}}{\underset{︸}{\text{v}\cdot \nabla c}}$$

where c is concentration, t is time, D is the diffusion coefficient, v is velocity field; and ∇ is the del operator.

Transport due to diffusion is controlled by the gradient in concentration at any given location and regulated by the diffusion coefficient of the analyte within the solvent. Transport due to advection is governed by the underlying flow field. This is determined through solution of a steady state Navier-Stokes Equation, subject to appropriate boundary conditions, which models the laminar fluid flow:

(3) $$\underset{\text{acceleration}}{\underset{︸}{\rho \text{v}\cdot \nabla \text{v}}}=\underset{\text{pressure gradient}}{\underset{︸}{-\nabla p}}+\underset{\text{viscous}}{\underset{︸}{\mu \nabla^{2}\text{v}}}\nabla \cdot \text{v}=0$$

where *ρ* is fluid density, p is fluid pressure, and *μ* is the coefficient of dynamic viscosity.

For this study, the underlying equations were solved using the finite element method [20] within Comsol Multiphysics (Comsol Inc., Burlington, MA, USA) This involved 3 key steps for solving the transport of a dilute species:

1. A 3-dimensional geometric model was built to match the experimental apparatus. The flow domain was represented by ∼100,000 tetrahedral elements.
2. The steady state flow equations were then solved subject to an appropriate set of boundary conditions: a volumetric flow inlet boundary condition (to match that within the experiment), a pressure boundary condition (0 Pa) at the outlet of the flow cell and a no-slip condition applied along the solid walls. The physical properties of the fluid were assumed to be those of pure water at 20 °C (density 998 kg/m^3^ and viscosity 1 × 10^−3^ Pa·s). The independence of the solution from the problem discretization was ensured by repeatedly solving the problem using a progressively finer computational mesh until a robust solution was obtained.
3. The advection-diffusion equation (Equation (2)) is solved subject to a velocity field specified from step 1, with a diffusion coefficient of 5 × 10^−10^ m^2^/s to match that of sodium fluorescein in water [15]. The initial concentration throughout the domain was set to 0, and at time zero the concentration at the inlet was set to c = 1. At the flow outlet transport is by advection only, whilst the walls are constrained to zero flux. As before, the independency of the solution on both the grid density and time step is confirmed by using progressively finer meshes and smaller time steps.

A flow rate range of 0.1–10 mL/min was simulated in the numerical models (*i.e.*, a wider range than considered in the experimental study).

## 3. Results and Discussion

**Figure 4.**
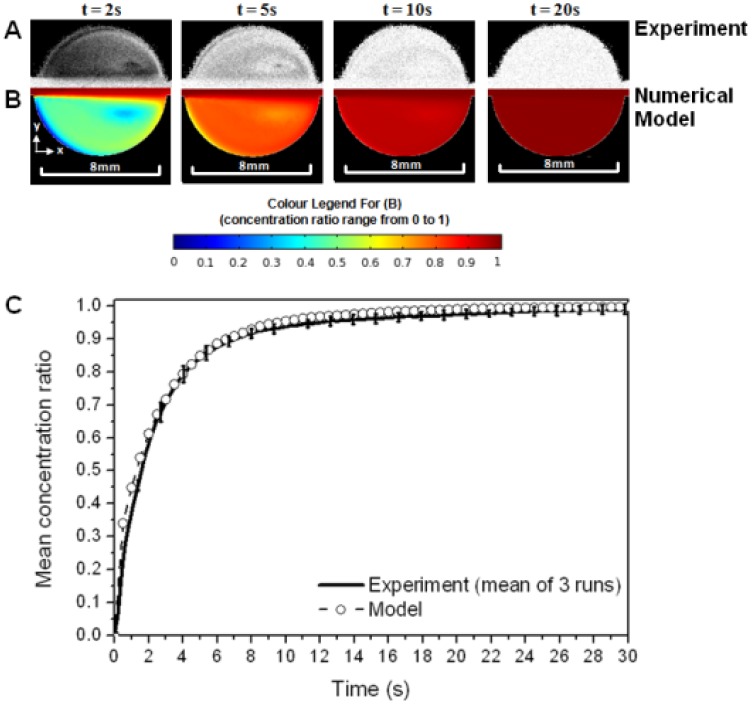
Performance of the circular flow cell when the flow rate was 10 mL/min: (**A**) processed experimental images, where black is low concentration and white is high concentration, (**B**) predicted concentration maps, and (**C**) Mean concentration ratio within the flow cell as a function of time.

Results for the circular cell design under a flow rate of 10 mL/min demonstrate that there is very good agreement between the experimental and computational techniques employed in this study (see Figure 4).

The concentration ratio maps in Figure 4(A,B) show that, qualitatively, the flow pattern evolves in a very similar manner with time. The variation in mean analyte concentration ratio with time (Figure 4(C)) shows that there is good quantitative agreement between the experimental and computational models. The error bars in Figure 4(C) (±1 standard deviation) show that the experimental model exhibited good repeatability (the error thus defined was never more than 3.5% of the mean concentration). Similar comparisons to those shown in Figure 4 were undertaken for a lower flow rate (1 mL/min) and for the other channel-flow geometries, and again good repeatability was found in the experimental runs and a good level of agreement is observed between the experimental and computational models.

**Figure 5.**
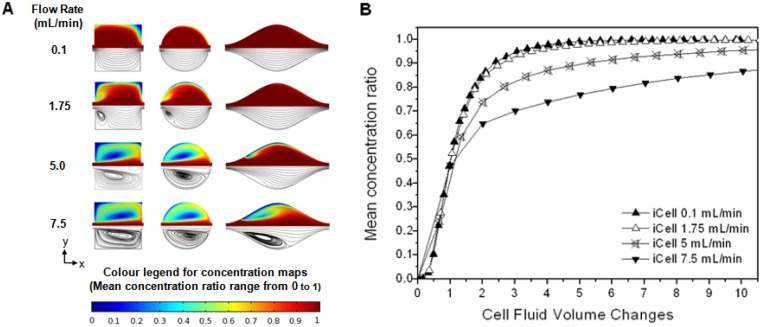
Computational results showing (**A**) concentration maps (top) and streamlines (bottom), after 5 flow cell volume changes and taken through the mid-plane for four selected flow rates; (**B**) Response of flow cell to injection of analyte for iCell at four selected flow rates.

The variation in the flow pattern with flow cell geometry and flow velocity was investigated using the numerical model (Figure 5). At low velocities (and hence *Re*) the flow followed the contours of the flow cell from inlet to outlet, but the development and growth of eddies was clearly observed as velocity (and hence *Re*) increased. In the circular cell and iCell eddies were not observed at low flow rates, but appeared at higher flow rates (∼1 and ∼2.5 mL/min respectively) and increased in size as the flow-rate further increased. These fit the definition of “inertial eddies” [21] which form due to flow separation above some critical flow rate. Eddies were present in the square cell at all the velocities investigated, but increased in size as the flow rate increased; these fit the definition of corner eddies [22,23].

The impact of eddy development on the mean analyte concentration ratio within a flow cell was explicitly considered for the iCell (Figure 5(B)). The responses at the two lower flow rates were very similar, where the flow follows the contours of the cell from inlet to outlet (Figure 5(A)), which resulted in a progressive increase in solute concentration along the length of the cell with time (see supplementary information for flow simulations). This pattern of flow indicates that advection is the dominant solute transport process. At higher flow rates there is a core of flow directly between the inlet and outlet along the centerline of the cell, but areas of recirculation either side. The solute concentration increased along this core (where advection is the dominant transport process), but far more slowly in the eddies. Solute transport into the eddies must be through diffusion. This is a significantly slower process than advective transport in the system under study, so the overall cell response at the two higher flow rates is significantly slower than the cases where eddies are not present. The same pattern of flow can also be observed for the circular cell (Figure 4(B,C)).

**Figure 6.**
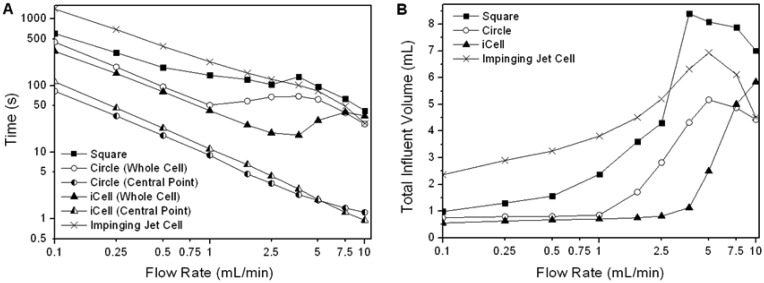
(**A**) Time taken and (**B**) required influent volume for concentration within flow cell to reach 99% of that of injected fluid (as a function of inlet flow rate). Also shown for (**A**) is the response for a single point at the centre of the flow domain for the iCell and the circular cell.

As the analyte concentration in the flow cell approaches the influent concentration asymptotically, the most appropriate way to evaluate the performance of a flow cell is to compare the time and/or flow volume required to reach some pre-determined proportion of the influent concentration in the cell (the cell concentration ratio, or CCR) over a range of flow-rates appropriate to practical operation. The performance of the three channel flow cells for a CCR of 99% and a range of flow-rates is shown in Figure 6 (the performance of a commercial impinging jet cell is shown for comparison). For all cell geometries there is initially a decrease in the time taken to reach the CCR as the flow rate increases (Figure 6(A)). However the flow volume required to reach the CCR in the circular and iCell initially remains constant (Figure 6(B)), demonstrating that advection is the dominant transport mechanism. Once eddies start to form the benefit of increasing the flow-rate is lost. Thus there is an optimal flow rate for these cells, at which both the time and flow volume require to reach the CCR are minimum. For the square cell corner eddies were present at all flow rates, and the flow volume required to reach 99% CCR increased with flow velocity (reflecting the increasing dominance of eddies). Once eddies were present, the flow volume required to reach the CCR increased for all the cells until the point where the eddies spanned the width of the flow cell (3.75, 5.0 and ∼15 mL/min in the square, circular and iCell, respectively), when some performance is recovered.

At all flow rates considered in this study some regions within any given cell will reach a given concentration of analyte quicker than others (e.g., locations aligned with the inlet respond more quickly than locations towards the edge of the cell). This is illustrated in Figure 6(A) which compares the concentration of the analyte at the centre point of the flow cell to the mean whole-cell response for the circular and iCell designs.

The performance of a commercially available impinging jet type flow cell (Figure 3(C)) is significantly poorer than either the circular cell or the iCell for all but the highest flow rate despite the cell having a smaller internal volume. Examination of the flow regime in detail revealed that there are large regions in this flow cell where advection has little impact and diffusion is the dominant transport process.

There are clear advantages to operating a flow cell close to the optimum point prior to eddy development, as the cell response will be independent of the diffusion coefficient of the analyte, and relatively insensitive to slight variations in flow rate. For the circular flow cell this optimum occurred at a flow rate of 1 mL/min, when the total influent volume was 0.85 mL and the injection time was 51 s. For the iCell the optimum was at a flow rate between 1.75 and 2.5 mL/min, when the total influent volume was between 0.75 and 0.81 mL and the injection time was between 26 and 20 s. Thus the iCell performed best out of the cell geometries considered, both in terms of the smallest influent volume required (least waste produced) and shortest injection time (the period for which the biosensor is exposed to a varying analyte concentration).

## 4. Implications for Sensor Operation

The selection of an appropriate operating protocol for a flow cell will depend on a proper understanding of the biosensor kinetics. The rates of ligand-receptor association (k_a_) and dissociation (k_b_) must be considered relative to the flow cell response rate. Where k_a_ and k_b_ are of similar magnitude whether fast or slow relative to the flow cell response rate, e.g., [24,25] the biosensor will equilibrate with the final cell concentration, and a robust measurement can be made. Therefore the main operational consideration is to ensure that the desired CCR is reached (e.g., 99% of influent concentration). A sensor with fast kinetics relative to the flow cell response rate will respond “instantaneously” to the cell concentration, but when the kinetics are slow e.g., [26] time for equilibration must be allowed once the cell reaches the desired CCR.

Sensors where ligand-receptor association is fast in comparison with ligand-receptor dissociation are more problematic. The ratio k_a_/k_b_ (equal to the distribution coefficient, K_d_) is a measure of how the analyte partitions between the sensor surface and the solution when equilibrium is reached. A high k_a_/k_b_ value implies more analyte association with the sensor for a given solution concentration than a low k_a_/k_b_ value. Thus high degrees of surface association, where the sensor becomes less sensitive to differences in solution concentration, can be reached with relatively modest solution concentrations. Operating this type of sensor with modest or high solution concentrations at a high CCR will give a small dynamic range. However, with better understanding of the flow dynamics within the cell it may be possible to operate such sensors as “accumulation sensors” where the flow cell is briefly operated for achievement of a lower CCR value, flow is ceased and the sensor is allowed to equilibrate. The solution concentration can then be estimated from the sensor response using a cell factor.

The final consideration when optimizing a flow cell operating protocol will be the operating environment. Automated systems which are to be deployed remotely in the field, for example, will be constrained by the need to minimize effluent volumes (since this may have to be stored, depending on local regulations) and power usage. Additionally, whilst this article is focused upon the operation of biosensors within a flow cell unit the same principles and derived implications can be applied to other sensor types such as chemical sensors (e.g., potentiometric ion selective electrodes).

## 5. Conclusions

The behavior of a flow cell, measured in terms of its response to the incoming fluid, is critically dependant on both the shape of the flow cell and the flow rate of the influent. A badly designed cell or one operated above its optimum flow rate can trigger recirculation (eddies) within the flow. Once this occurs, the time and number of volume changes required to reach some predefined proportion of the influent analyte concentration (the cell concentration ratio—CCR) can be larger than that where fluid injection takes place at a lower flow rate. More importantly, outside the optimum range of flow-rates the required number of volume changes to achieve the CCR becomes more sensitive to the flow-rate used.

A range of flow cell geometries have been studied and it has been demonstrated that a flow cell with a smooth profile between the inlet and outlet (the iCell) outperformed (in terms of time to reach the CCR and total flow volume) those flow cells based on circular or square profiles or a commercial impinging jet type design, despite the iCell having the largest internal volume.

Key recommendations for the design and operation of biosensor flow cell systems are:

- Select a flow cell design and operational protocol that prevents the development of flow recirculations (eddies) to minimize the volume of fluid and time to reach the CCR. This can be achieved by the combined effect of reducing the rate of channel expansion and contraction, and maintaining a flow rate below which the onset of eddies arise.
- In all cases the response of a given flow cell is sensitive to flow-rate and flow domain geometry and thus good flow control is a pre-requisite for repeatable and efficient sensing.
- To achieve accurate measurements using biosensors it is important for users to understand that (a) the concentration of analyte within the flow cell will differ from that within the influent, and (b) the time required to reach the CCR is a function of the cell geometry as well as the flow-rate, and thus the flow cell performance must be fully characterized before use.

## Electronic Supplementary Information

Figure S1 and Videos SV1–SV4.

Supplementary File 1 Supplementary Information (PDF, 151 KB)

Supplementary File 2 Video SV1 (full details listed in Supplementary Information PDF document above) (AVI, 17042 KB)

Supplementary File 3 Video SV2 (full details listed in Supplementary Information PDF document above) (AVI, 6720 KB)

Supplementary File 4 Video SV3 (full details listed in Supplementary Information PDF document above) (AVI, 3532 KB)

Supplementary File 5 Video SV4 (full details listed in Supplementary Information PDF document above) (AVI, 3385 KB)
