# Supplementary material for: Flow Cell Design for Effective Biosensing
Source: Sensors (Basel). 2012 Dec 20;13(1):58–70. doi: 10.3390/s130100058 (PMC3574664; doi:10.3390/s130100058)
Supplement: Supplementary File 1 — Supplementary Information (PDF, 151 KB) [file sensors-13-00058-s001.pdf]

OPEN ACCESS

sensors

ISSN 1424-8220

www.mdpi.com/journal/sensors

Supplementary Information

Flow Cell Design for Effective Biosensing. *Sensors* 2013, *13*, 58-70

Douglas J. Pike [1,*], Nikil Kapur [2], Paul A. Millner [3] and Douglas I. Stewart [1]

1 Pathogen Control Engineering (PaCE) Institute, School of Civil Engineering, University of Leeds, Leeds, West Yorkshire, LS2 9JT, UK; E-Mail: d.i.stewart@leeds.ac.uk

2 Institute of Engineering Thermofluids, Surfaces & Interfaces (iETSI), School of Mechanical Engineering, University of Leeds, Leeds, West Yorkshire, LS2 9JT, UK; E-Mail: n.kapur@leeds.ac.uk

3 Institute of Membrane and Systems Biology, Faculty of Biological Sciences, University of Leeds, Leeds, West Yorkshire, LS2 9JT, UK; E-Mail: p.a.millner@leeds.ac.uk

* Author to whom correspondence should be addressed; E-Mail: ee09djp@leeds.ac.uk; Tel.: +44-11-3343-2399.

Modelled Flow Animations

Four animation videos are presented (alongside this document) that show flow simulations for the circular flow cell design at flow rates of 0.1, 1.75, 5.0 & 7.5 mL/min (Videos SV1–SV4), taken as a plan-view through the mid-plane of the flow cell. Flow cell dimensions on each video are shown in mm. These videos directly supplement Figure 5(A) of the main paper which presented snapshot images after five flow cell volume changes for the same flow conditions. The response of the circular flow cell at the four selected flow rates is presented in Figure 1(A).

Video Details

Video SV1 Flow simulation animation for circular flow cell design at a flow rate of 0.1 mL/min. Animation is based upon numerical solutions at 1s intervals and an output rate of 15 frames per second. Video length is 33.3 s.

Video SV2 Flow simulation animation for circular flow cell design at a flow rate of 1.75 mL/min. Animation is based upon numerical solutions at 1s intervals and an output rate of 15 frames per second. Video length is 13.3 s.

Video SV3 Flow simulation animation for circular flow cell design at a flow rate of 5 mL/min. Animation is based upon numerical solutions at 1s intervals and an output rate of 15 frames per second. Video length is 6.7 s.

Video SV4 Flow simulation animation for circular flow cell design at a flow rate of 7.5 mL/min. Animation is based upon numerical solutions at 1s intervals and an output rate of 15 frames per second. Video length is 6.7 s. Flow cell dimensions are shown in mm.

Figure S1. Computational results showing response of flow cell to injection of analyte (scaled between 0—no analyte and 1—analyte concentration within cell equal to that of injected fluid) for circular cell at four selected flow rates.

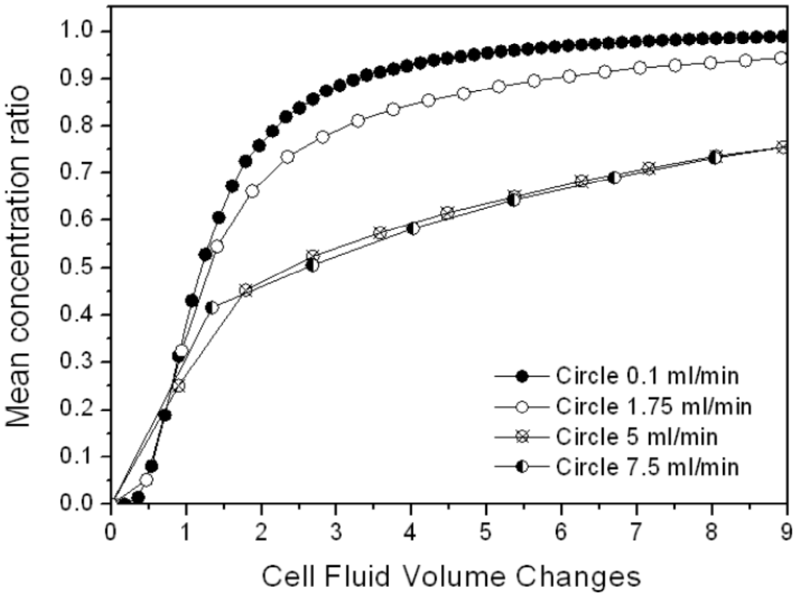

© 2013 by the authors; licensee MDPI, Basel, Switzerland. This article is an open access article distributed under the terms and conditions of the Creative Commons Attribution license (http://creativecommons.org/licenses/by/3.0/).
